# Pericardial immunoglobulin G4‐related inflammatory pseudotumor after right upper lobectomy for lung cancer

**DOI:** 10.1111/1759-7714.13633

**Published:** 2020-08-26

**Authors:** Risa Oda, Katsuhiro Okuda, Takayuki Murase, Tadashi Sakane, Tsutomu Tatematsu, Keisuke Yokota, Katsuhiko Endo, Ryoichi Nakanishi

**Affiliations:** ^1^ Department of Oncology Immunology and Surgery, Nagoya City University Graduate School of Medical Sciences Nagoya Japan; ^2^ Department of Pathology and Molecular Diagnostics Nagoya City University Graduate School of Medical Sciences Nagoya Japan

**Keywords:** Immunoglobulin G4‐related (IgG4‐related) inflammatory pseudotumor, lung cancer, pericardial tumor

## Abstract

A 75‐year‐old woman underwent thoracoscopic right upper lobectomy for lung cancer. A histopathological examination showed adenocarcinoma, pT1aN0M0 stage IA1. At six months after surgery, chest computed tomography (CT) revealed pericardial nodules that had not been detected before pulmonary resection. Postoperative CT performed two months later revealed that the nodules were growing and F^18^ fluorodeoxyglucose‐positron emission tomography showed a maximum standardized uptake of 9.87. Blood tests revealed no elevated tumor markers, with the exception of a mildly elevated interleukin‐2. Based on the above results, thoracoscopic biopsy was performed due to the suspected recurrence of lung cancer or malignant lymphoma. The histopathological examination of the nodule revealed immunoglobulin G4 (IgG4)‐related inflammatory pseudotumor. The serum IgG4 levels were elevated (358 mg/dL, normal: 4.5–117.0 mg/dL). No additional treatment was required because all nodules were observed to have disappeared naturally on a follow‐up CT scan performed two months after the surgical biopsy. The patient has been followed‐up for two years without recurrence.

**Key points:**

**Significant findings of the study:**

We report a case of pericardial immunoglobulin G4‐related inflammatory pseudotumor that appeared after right upper lobectomy for lung cancer, and which naturally disappeared without any treatment.

**What this study adds:**

There was an immunoglobulin G4‐related inflammatory pseudotumor which appeared as multiple nodules in the pericardial space, and this should be kept in mind when considering the differential diagnosis of intrapericardial nodules.

## Graphical abstract

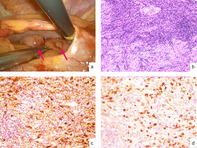

## Introduction

Immunoglobulin G4 (IgG4)‐related disease can involve multiple organs, particularly exocrine organs, such as the pancreas, salivary glands, and biliary tract,^1^, ^2^ but is relatively rare in the pericardium. Although there have been reports of IgG4‐related disease with pericardial thickening and pericardial effusion,^3^, ^4^, ^5^, ^6^ our search of the relevant literature revealed no cases with intrapericardial nodules.

We herein report a case of IgG4‐related inflammatory pseudotumor with pericardial nodules after right upper lobectomy for lung cancer.

## Case report

**Figure 1:**
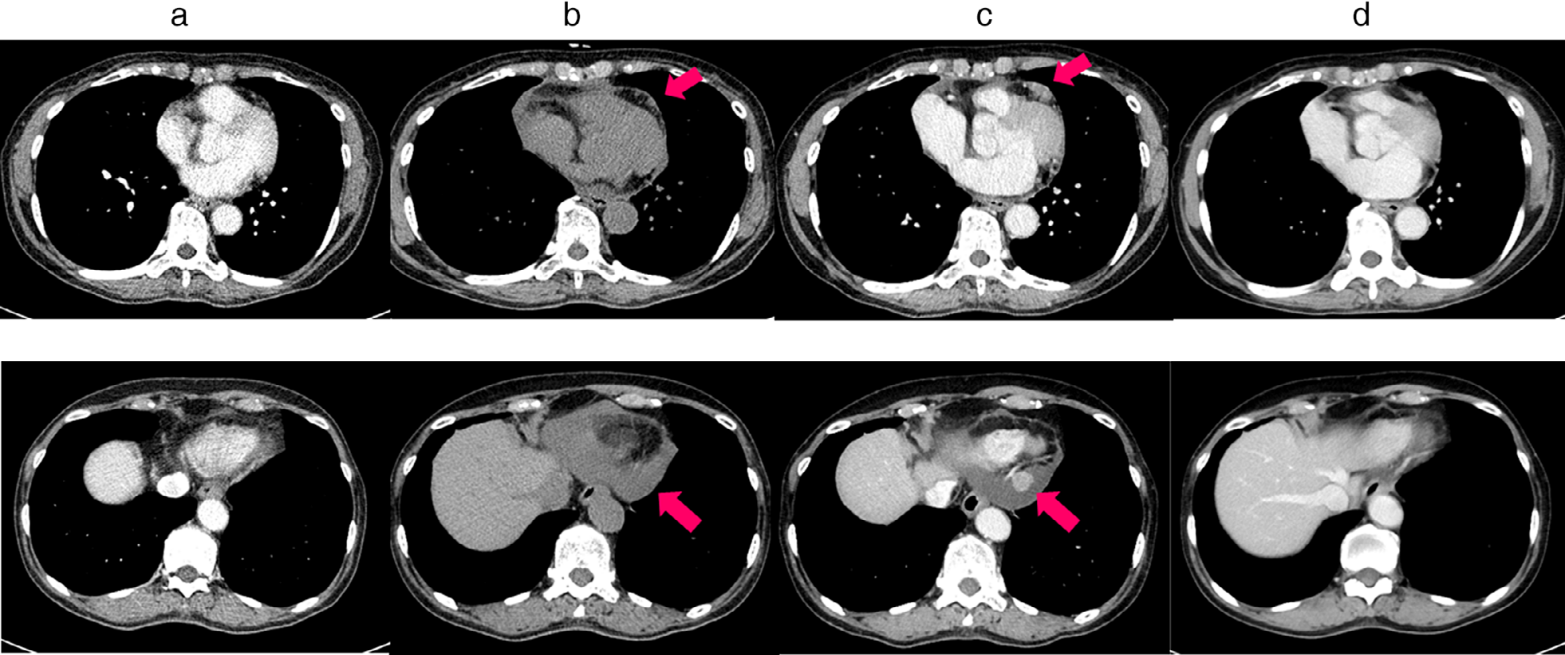
(**a**) The upper image shows computed tomography of the chest showing 7 cm below the carina. The lower image shows 1 cm above the pericardial end. There were no nodules in the pericardium before lung cancer surgery. (**b**) Several small nodules were observed in the pericardial space at six months after lung cancer surgery. (**c**) The nodules grew and pericardial effusion increased at eight months after lung cancer surgery. (**d**) All nodules had naturally disappeared at two months after surgical biopsy.

**Figure 2:**
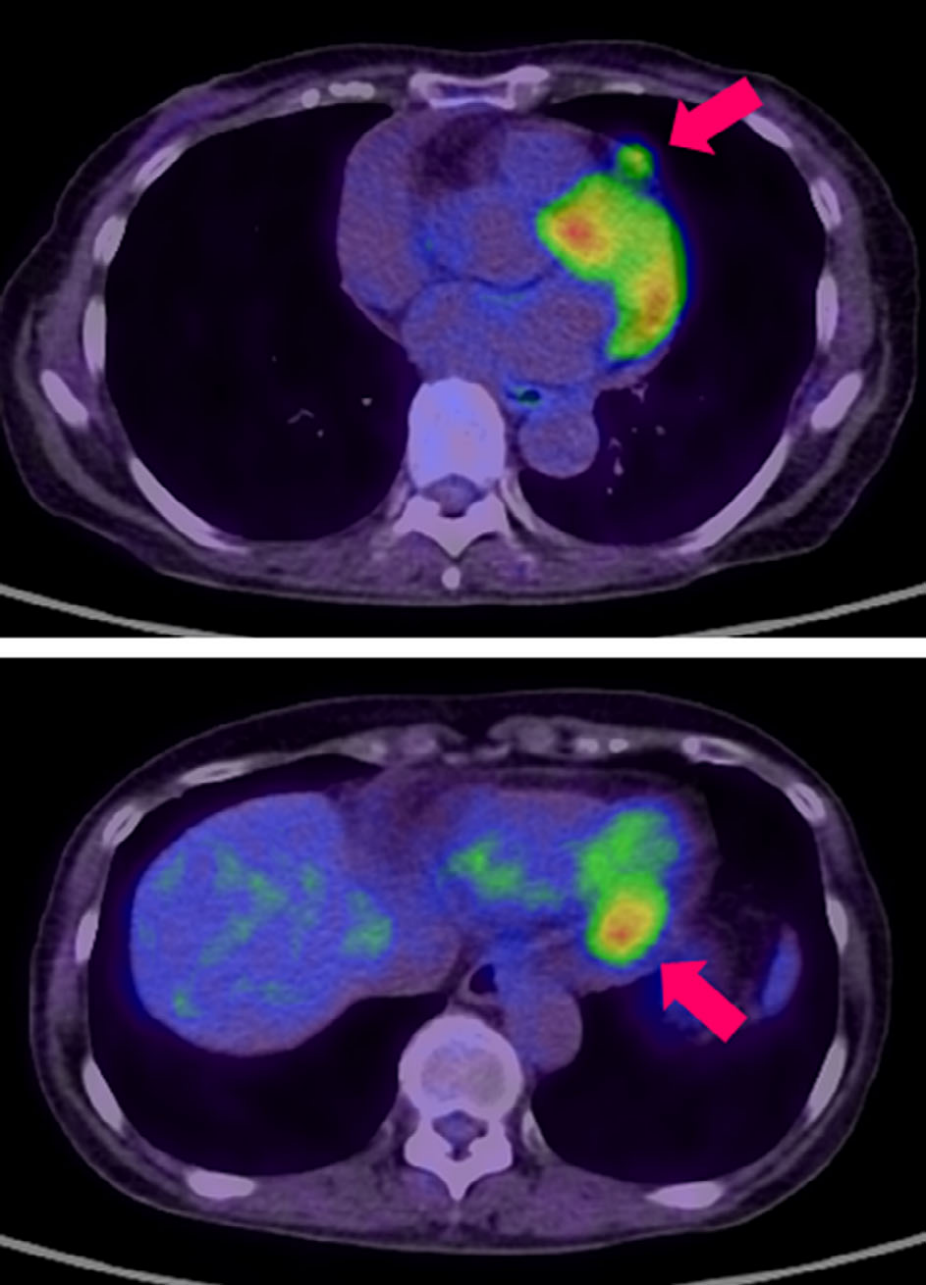
F^18^ fluorodeoxyglucose‐positron emission tomography showed a high uptake in the pericardial nodule at eight months after lung cancer surgery.

A 75‐year‐old woman underwent thoracoscopic right upper lobectomy for lung cancer. The histopathological diagnosis showed adenocarcinoma, pT1aN0M0 stage IA1 according to the eighth edition of the TNM lung cancer staging system.^7^ Although preoperative chest computed tomography (CT) and echocardiography did not reveal any nodules in the pericardium (Fig 1a), regular follow‐up CT at six months after surgery revealed pericardial nodules and pericardial effusion (Fig 1b). On CT scan performed two months later, the nodules had grown and pericardial effusion had increased (Fig 1c). F^18^ fluorodeoxyglucose‐positron emission tomography (FDG‐PET) showed a maximum standardized uptake value of 9.87 (Fig 2). At this time, the patient's laboratory data were normal, including the levels of tumor markers associated with lung cancer, with the exception of an elevated level of soluble interleukin‐2 (IL‐2) receptor at 805 U/mL (normal: 145–519 U/mL).

**Figure 3:**
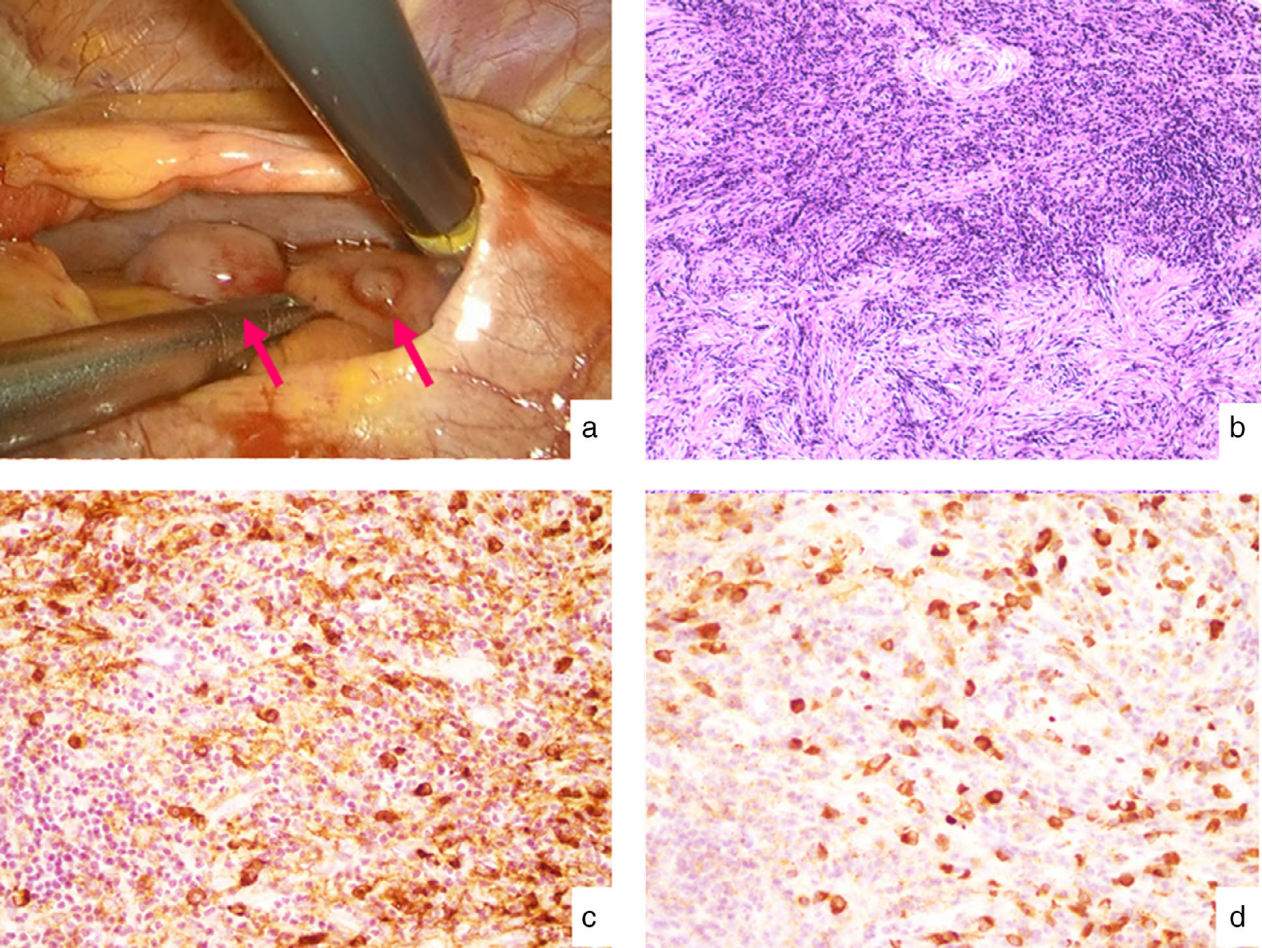
(**a**) An intraoperative view of the nodules within the pericardial sac. (**b**) Lymphoplasmacytic infiltration and storiform fibrosis with lymphoid follicles were observed on hematoxylin and eosin staining (original magnification ×200). (**c**) Immunohistochemical staining for immunoglobulin G4; and (**d**) G (original magnification ×200). The IgG4/IgG‐positive cell ratio was 40%.

We suspected malignant lymphoma or recurrence of lung cancer. We planned surgical biopsy of the pericardial nodules by video‐assisted thoracoscopic surgery VATS). The operation was performed in the right lateral decubitus position with three ports via the left thoracic cavity. The nodules were found within the pericardial sac (Fig 3a); two nodules were obtained as specimens.

The final histopathological examination of the nodules revealed IgG4‐related inflammatory pseudotumor. Numerous IgG4‐positive cells were found on immunostaining and the ratio of IgG4 staining to total IgG staining was approximately 40% (Fig 3b–d). The patient's serum IgG4 level after surgery was elevated at 358 mg/dL (normal: 4.5–117.0 mg/dL). We consulted the Department of Allergy and Clinical Immunology after the patient had been diagnosed with IgG4‐related inflammatory pseudotumor. Based on their advice, we did not provide any additional treatment because the patient did not complain of any symptoms and all of the nodules disappeared naturally on a follow‐up CT scan performed two months after the surgical biopsy (Fig 2d). Although the patient's serum IgG4 levels and soluble IL‐2 receptor after two years were still elevated at 690 and 1150, respectively, the patient was followed‐up for two years without recurrence.

## Discussion

IgG4‐related disease can involve virtually every organ. The most frequently involved sites are the pancreas, bile ducts, gallbladder, liver, salivary glands and kidneys, with manifestations generally recognized as a mass at one or more sites mimicking a neoplasm.^2^ In the thoracic region, IgG4‐related disease manifests as interstitial lung disease. In a Japanese study of 114 patients with IgG4‐related disease, 16 (14.0%) patients had pulmonary involvement, and none had pericarditis.^3^ There have been some reports of occurrence in the pericardium.^4^, ^5^, ^6^ These studies reported pericardial thickening or effusion. No cases of pericardial nodules have been reported as IgG4‐related disease.

In the present case, the nodules found in the pericardium were growing during two months of follow‐up; we therefore suspected the recurrence of lung cancer and resected the pericardial nodules. However, considering that the pericardium was not treated at the time of right upper lobectomy and that the pericardial nodules that appeared six months after surgery for lung cancer had a higher SUV max than the primary lung cancer (SUV max 1.55), it was possible to follow‐up the patient with CT scan rather than perform a risky surgical biopsy. There are few reports regarding pericardial events after lung cancer surgery, other than recurrence. In these cases, the reported causes of pericardial effusion include residual undrained blood and the prevention of lymphatic drainage after mediastinal dissection.^8^, ^9^

It is difficult to obtain a correct diagnosis of pericardial tumor. In this report, we failed to consider IgG4‐related disease as a differential diagnosis before surgical biopsy and surgical resection was required to make a correct diagnosis based on a histopathological examination. Some reports have described elevated serum levels of soluble IL‐2 receptor in cases of IgG4‐related disease.^6^, ^10^ The mildly elevated levels of soluble IL‐2 receptor in the present case may have therefore been suggestive of IgG4‐related disease. If we had focused on the results of FDG‐PET and soluble IL‐2 receptor, this might have led us to strongly suspect inflammatory disease such as IgG‐4‐related disease, thereby avoiding a potentially risky surgical biopsy.

Previous studies have proposed that FDG‐PET is superior to other imaging modalities for the diagnosis of IgG4‐related disease. Several reports have shown the abnormal uptake of the tumor; however, there was no significant difference in abnormal uptake between IgG4‐related disease and other malignant tumors. FDG‐PET can define more lesions than conventional imaging methods by providing the whole‐body metabolic condition and morphological abnormality, and it can serve as a sensitive tool in assessing organ involvement and disease distribution.^11^, ^12^ In our case, FDG‐PET showed an abnormal uptake that was higher than the primary lung cancer, and the pericardial nodules grew after only two months. Based on the findings of the PET scan and rate of tumor growth, we should have considered inflammatory disease, especially IgG4‐related disease, as a differential diagnosis.

To our knowledge, there are no previous reports on IgG4‐related disease with pericardial nodules. Based on this case report, it was shown that IgG4‐related disease should be included in the differential diagnosis of pericardial nodules and it might be important to measure serum soluble IL‐2 receptor.

Although IgG4‐related disease in the pericardium is rare, we should consider this disease in the differential diagnosis of patients with pericardial nodules.

## Disclosure

The authors have no conflicts of interest to declare.

## References

[1] Brito‐Zerón P , Ramos‐Casals M , Bosch X , Stone JH . The clinical spectrum of IgG4‐related disease. Autoimmun Rev 2014; 13: 1203–10. 25151972 10.1016/j.autrev.2014.08.013

[2] Islam AD , Selmi C , Datta‐Mitra A e a . The changing faces of IgG4‐related disease: Clinical manifestations and pathogenesis. Autoimmun Rev 2015; 14: 914–22. 26112170 10.1016/j.autrev.2015.06.003

[3] Zen Y , Nakamura Y . IgG4‐related disease: A cross‐sectional study of 114 cases. Am J Surg Pathol 2010; 34: 1812–9. 21107087 10.1097/PAS.0b013e3181f7266b

[4] Sugimoto T , Morita Y , Isshiki K *et al* Constrictive pericarditis as an emerging manifestation of hyper‐IgG4 disease. Int J Cardiol 2008; 130: 100–1. 10.1016/j.ijcard.2007.06.111 17727980

[5] Sekiguchi H , Horie R , Utz JP , Ryu JH . IgG4‐related systemic disease presenting with lung entrapment and constrictive pericarditis. Chest 2012; 142: 781–3. 22948582 10.1378/chest.11-2608

[6] Morita T , Izawa A , Hamano H *et al* Significant pericardial involvement of immunoglobulin G4‐related disease. Ann Thorac Surg 2014; 98: 47–9. 10.1016/j.athoracsur.2014.04.069 25087832

[7] Goldstraw P , Chansky K , Crowley J e a . The IASLC lung cancer staging project: Proposals for revision of the TNM stage groupings in the forthcoming (eighth) edition of the TNM classification for lung cancer. J Thorac Oncol 2016; 11: 39–51. 26762738 10.1016/j.jtho.2015.09.009

[8] Tomimaru Y , Kodama K , Okami J , Oda K , Takami K , Higashiyama M . Pericardial effusion following pulmonary resection. Jpn J Thorac Cardiovasc Surg 2006; 541: 93–198. 10.1007/BF02670311 16764307

[9] McClean RH , Parandian BB , Nam MH . Pericardial tamponade: An unusual complication of lobectomy for lung cancer. Ann Thorac Surg 1999; 67: 545–6. 10197691 10.1016/s0003-4975(98)01245-4

[10] Sato Y , Notohara K , Kojima M , Takata K , Masaki Y , Yoshino T . IgG4‐related disease: Historical overview and pathology of hematological disorders. Pathol Int 2010; 60: 247–58. 20403026 10.1111/j.1440-1827.2010.02524.x

[11] Zhang J , Chen H , Ma Y e a . Characterizing IgG4‐related disease with ^18^F‐FDG PET/CT: A prospective cohort study. Eur J Nucl Med Mol Imaging 2014; 41: 1624–34. 24764034 10.1007/s00259-014-2729-3 PMC4089015

[12] Berti A , Della‐Torre E , Gallivanone F e a . Quantitative measurement of 18F‐FDG PET/CT uptake reflects the expansion of circulating plasmablasts in IgG4‐related disease. Rheumatology (Oxford) 2017; 56: 2084–92. 28977663 10.1093/rheumatology/kex234

